# Decision-making at the crime scene: A scoping literature review on the cognitive aspects of crime scene examination and the influence of rapid testing

**DOI:** 10.1016/j.fsisyn.2026.100749

**Published:** 2026-10-27

**Authors:** Timon G. Udo, Jasper J. van der Kemp, Christianne J. de Poot

**Affiliations:** a Vrije Universiteit Amsterdam, Department of Criminology, De Boelelaan 1105, Amsterdam, 1081 HV, Netherlands; b Police Academy of the Netherlands, Knowledge & Research Department, Arnhemseweg 348, Apeldoorn, 7334 AC, Netherlands; c Amsterdam University of Applied Sciences, Forensic Investigations, Tafelbergweg 51, Amsterdam, 1105 BD, Netherlands

**Keywords:** Rapid tests, Crime scene examination, Decision-making, Cognitive factors, Crime scene investigators

## Abstract

Rapid tests will likely become increasingly available to crime scene investigators and have the potential to fundamentally change how decisions are made during the examination of the crime scene. By speeding up the analysis of traces, preliminary results from on-site rapid testing can already be used to support decision-making at the crime scene and may ultimately transform how crime scenes are examined. However, crime scene investigators' decision-making remains influenced by cognitive factors that also affect current examination practices. Given the lack of practical guidance on decision-making in guidelines and forensic science handbooks, it is essential to understand how crime scene investigators make decisions in practice. In this scoping literature review (N = 32), we examine how crime scene investigators search for, interpret, collect, and analyse traces, and how the introduction of rapid tests may influence these decision-making processes. The review reveals that guidelines and handbooks have paid little attention to cognitive factors that may shape investigators’ observations and decisions. Empirical studies, however, show that cognitive factors play an important role in the decision-making at the crime scene. Understanding these influences is becoming increasingly important as rapid testing may affect early decision-making and, in turn, may iteratively shape the course of the investigation. Further research is therefore needed to better understand how rapid testing may influence decision-making at the crime scene.

## 1 Introduction

Technological advances have led to techniques that allow for rapid on-site analysis of forensic traces (see, for example, [1], [2], [3], [4]). In time, these rapid techniques may become available to crime scene investigators, which would allow them to already use the results of rapid analyses during their examination of the crime scene. This is in stark contrast to the current situation where the results of conventional analyses typically only become available after traces have been collected and submitted to a laboratory. Although innovations have also reduced the time laboratories need to produce results, in the Netherlands only highly urgent biological traces suitable for DNA analysis can be analysed and compared with the DNA database within hours. For most other DNA-bearing traces, this typically takes three days, provided that the traces are already sampled by the police and immediately submitted to the laboratory [5]. Similarly, it takes two working days for the results of toxicological analyses to become available [6]. Because conventional trace analysis typically takes days, except for highly urgent traces, its outcomes often cannot be used to inform decision-making at the crime scene. As a result, decisions made during the crime scene examination are based on limited information. The rapid nature of new techniques may, to some extent, change this by providing additional information at an early stage, helping investigators better understand what may have happened and which traces may warrant collection. Although rapid testing may, accordingly, change how crime scenes are examined, its use is likely to interact with existing practices. Current practices inform search strategies and will continue to influence where crime scene investigators initially search for traces and which traces become available for rapid analysis. Within this process, rapid tests may support the detection or visualisation of traces, thereby, influencing which traces are noticed and which information becomes available. Moreover, rapid tests that provide additional information about the traces can further influence which information is available and how information is interpreted. This may, in turn, influence where crime scene investigators search for additional traces and which traces they decide to collect and analyse. Rapid testing may therefore create a continuous feedback loop during crime scene examination, whereby preliminary results inform subsequent search and recovery activities, enabling investigators to target and rapidly analyse the most relevant traces. As the influence of rapid testing may thus become intertwined with current practices of crime scene examination and decision-making, understanding the interaction between current practices and the use of rapid tests is essential to anticipate consequences of implementation in practice. Although practical implementation may depend on other factors as well, such as operational or organisational constraints and the quality of the rapid test, the cognitive processes underlying crime scene examination warrant particular attention. Rapid testing introduces new information into an examination that is already shaped by investigators’ expectations, attention, interpretations, and decisions. How this new information is noticed, interpreted, and incorporated into subsequent decisions may therefore influence how crime scenes are searched, which traces are recognised as relevant, and which traces are selected for further examination. It is therefore imperative to understand how crime scene investigators currently find, recognise, collect, and analyse traces and to explore how the availability and use of rapid tests may influence this, in order to identify which questions need to be answered before rapid testing can be implemented. We therefore first discuss the phases of crime scene examinations and the cognitive aspects and decision-making that play a role in these examinations, before turning to the empirical literature on how crime scene investigators examine crime scenes and how rapid testing appears to influence these processes.

### 1.1 Crime scene examination phases

International guidelines describe the examination of crime scenes as a process consisting of several phases. Upon arrival at the crime scene, crime scene investigators should first gather the available information on potential safety hazards, the integrity of the crime scene, and potential witnesses. They should also assess whether the demarcation of the crime scene should be adjusted [7], [8].^1^ This information should then be used to develop a strategy for entering the scene and conducting an initial assessment [7].

The second phase involves an initial assessment of the crime scene, which should guide subsequent logistical and practical decisions, such as the route to be taken and the equipment and expertise required. This phase should also be used to establish investigative priorities [7], [8]. General photographs of the crime scene should then be taken, and traces that are visible at first glance or areas believed to contain traces should be marked for later collection. Based on this initial assessment, a forensic strategy must be developed for the exhaustive search for and collection of traces in the subsequent phase [7]. This strategy includes decisions about the appropriate search method(s), the sequence of examinations, which objects should be collected and how they can be collected safely while minimising the risk of contamination [8]. This strategy may be reassessed when additional information becomes available.

The third phase consists of the exhaustive search for and collection of traces. According to the guidelines of the European Network of Forensic Science Institutes (ENFSI), the search for traces should be conducted “[…] systematically and thoroughly for the relevant materials, targeting and prioritising areas, which in context of what has been alleged, are most likely to yield significant material of evidential value.” (p. 27) [7]. However, the ENFSI guidelines do not provide guidance on how investigators should identify the area's most likely to yield relevant traces. It only specifies a search order, denoting that crime scene investigators should first search systematically for visible traces, then for invisible traces using appropriate equipment such as forensic light sources, and finally for traces in less obvious places, such as inside furniture [7]. The guidelines also do not specify how a systematic search should be conducted. More concrete search strategies are described in forensic science handbooks. For example, investigators may divide the crime scene into grids and search each grid by moving horizontally and vertically through those grids (e.g., Ref. [9]). Alternatively, they may follow a path from the entrance point to the victim and subsequently to the exit [10], [11]. The systematic nature of these approaches is assumed to ensure that all traces are recognised, although this assumption is rarely made explicit: “Crime scene search patterns may vary but they share a common goal of providing organization and a systematic structure to ensure that no items of physical evidence are missed or lost” (p. 177) [12]. A similar approach should be applied outside the area demarcated by crime scene tape and may provide grounds for adjusting the boundaries of the crime scene. All traces identified within and outside the original boundaries should subsequently be collected [7].

Finally, the fourth phase involves reviewing the examination to determine whether its objectives have been achieved and whether additional examinations are required [8]. Upon completion of the examination, the investigative authorities should be informed of its results and the collected traces [7]. Afterwards, the examination should be documented in a report, which can be shared with other actors.

The introduction of rapid tests may fundamentally change these phases, where this review considers analytical techniques that can produce information about a forensic trace in time for the results to be used during the examination of the crime scene as a rapid test. Such techniques introduce additional decision steps about whether, when and how a rapid test should be used, while the results of such tests may also inform subsequent decision-making. For example, a rapid test indicating the possible presence of gunshot residue (e.g., Ref. [13]) could inform the decision whether a location should be included within the boundaries of the crime scene. During the initial assessment, a Kastle-Meyer test may indicate whether a red stain is likely to be blood (e.g., Ref. [14]), potentially affecting the subsequent forensic strategy. Similarly, indicative testing of whether a sample contains sufficient DNA for DNA profiling (e.g., Ref. [15]) may influence decisions about which traces to collect. Rapid DNA profiling may further influence the interpretation of traces and the crime scene more broadly. Although rapid testing may thus encompass a wide range of analytical techniques that could be useful at various stages and could each have their own user requirements, psychometric qualities and practical obstacles before implementation, they all provide additional information into the examination of the crime scene. As such, it seems plausible that rapid testing would not merely introduce an additional decision step concerning whether to use a rapid test but may rather influence the entire crime scene examination as well as subsequent stages of the criminal investigation.

### 1.2 Cognition and decision-making in crime scene examination

#### 1.2.1 Uncertainty in decision-making

As described above, crime scene examinations typically start with gathering information. However, available information is often limited and its relevance unclear. Consequently, multiple causes, explanations, and scenarios may be possible, requiring decisions to be made under conditions of uncertainty, which Beachler et al. [16] describe in terms of (maximum) entropy. Because crime scene investigators may initially not know what to look for or which information will prove relevant [16], fully rational decision-making would require the consideration of all possible scenarios and their consequences. However, Simon's [17] concept of bounded rationality suggests that humans are unable to do so, because of limited cognitive capacities. Instead, they often make decisions that are satisfactory and workable, rather than rationally optimal, a process Simon termed "satisficing". This perspective can be complemented by Kahneman's [18] distinction between two modes of cognitive processing: a deliberate, controlled and relatively slow mode of processing, and an intuitive, automatic and fast mode of processing. Given cognitive limitations and time constraints, humans often rely on the intuitive system, using heuristics – simple rules of thumb – and experience to make decisions [19], [20]. These heuristics can be useful because they enable quick decision-making, but they can also lead to errors or bias when they lead decision makers to rely on incomplete information or premature interpretations [21], [22].

#### 1.2.2 Finding traces

At the crime scene, the forensic investigation mainly consists of searching for and collecting traces. Although the ISO-norms and ENFSI-guidelines describe this process, they provide limited practical guidance on how traces should be identified and prioritised. By largely leaving these decisions unaddressed, these guidelines seem to either implicitly frame trace detection as a neutral and objective process or seem to remain at a high abstraction level resulting in limited practical guidance. After all, it has long been established that perception is selective and shaped by observers’ expectations, goals and prior knowledge and attentional resources (see Refs. [23], [24] for a review). An experiment showed, for instance, that 20 out of 24 experienced radiologists failed to notice a gorilla in a lung-nodule detection task while examining chest CT-scans, even though the gorilla was 48 times larger than the average nodule and eye-tracking data showed that most radiologists looked directly at its location [25]. Drew et al. hypothesised that the radiologists may have missed the gorilla because it did not resemble the lung nodules they were searching for. This study demonstrates that even experts can fail to notice abnormal stimuli that they looked at directly. Noticing stimuli or traces, thus, requires more than simply looking everywhere. This issue is further exacerbated by the increasing ability to collect and analyse smaller traces [26], including traces that are not visible to the naked eye. Visual cues alone are therefore insufficient to guide the search for traces. Instead, crime scene investigators must actively search for them, for example by applying and subsequently removing powder to a surface to reveal potential fingermarks [27], or blindly sample for invisible traces like touch DNA or saliva. Because not every surface can be powdered or sampled, investigators must identify and prioritise specific areas for examination rather than rely solely on systematic search patterns.

The assertion that systematic searches ensure that no relevant traces will be missed or overlooked, is furthermore problematic because recognising traces requires an interpretative step beyond merely attending to a trace. Stimuli, and therefore also traces, are not perceived objectively, as demonstrated, for example, by research on visual illusions (e.g., Refs. [28], [29]). Differences in perception are thought to arise from processes of unconscious inference, whereby the brain infers what real-world events gave rise to the sensory stimulation it receives. This process allows expectations based on prior experience to shape perception [30]. Such expectations may exert a particularly strong influence when stimuli are weak, noisy or ambiguous [23], as is often the case at crime scenes. In contrast, expectations are less likely to influence perception when stimuli are unambiguous or when expectations are highly inaccurate [23]. Therefore, although systematically structured searches may reduce the risk of traces being missed, it cannot guarantee their recognition. This is particularly relevant because investigators may not know in advance which traces will prove relevant to the case or how many traces, if any, are present.

#### 1.2.3 Relevance of traces

Instead of merely conducting a systematic search, Hazard [31] argues that crime scene investigators should use their (preliminary) knowledge of the case and of offender behaviours to formulate hypotheses about offender activities that can guide their search for traces. For each potential trace, crime scene investigators should consider: (1) the kind of source to which a trace relates, (2) its cause given the specific context of the case, and (3) the account that can be constructed from these observations. To then determine whether the trace is relevant for the investigation, two dimensions of relevancy should be considered: The first is factual or circumstantial relevancy, which concerns the hypothesised link between the trace and the case. The second is appropriateness relevancy, which concerns whether the resources required to collect and analyse the trace are justified by the expected outcome [31]. The latter dimension of relevancy appears to have some conceptual overlap with Bitzer's [32] concept of the utility of the clue, where utility refers to the added value of the information obtained through the analysis of a trace. She argues that the decision to analyse a trace is influenced by five knowledge dimensions that can function as a set of constraints or facilitators, where she positions the utility of the clue as the fifth dimension alongside the original four knowledge dimensions from Ribaux et al. [33]: the strategic dimension that includes aspects at an organizational level, the immediate dimension that constitutes aspects related to the specific case, the physical dimension that comprises characteristics of the trace, and the criminal dimension that relates to the knowledge of a crime link. For a trace to add value, it must be related to the activity under investigation, be understood by the crime scene investigator, and provide information that addresses a question in the case without being redundant. Although not originally conceptualised as such, this concept could also apply to trace collection, where traces that are expected to add value to the investigation are deemed relevant for collection.

Both concepts are, however, constrained by the limited information available when assessing either relevancy or expected utility. For the concept of utility of the clue, this is especially relevant when applied to trace collection. Hazard [31] acknowledges that it may not always be possible to establish a factual link between a trace and the case at this stage. Assessments of factual or circumstantial relevancy should therefore remain open to revision. Similarly, Bitzer [32] describes an *a priori* expected utility, which requires investigators to assess both factual and appropriate relevancy in the light of the case-specific information available at the time. As such, assessments of the expected utility are also limited by the lack of available information. Although Bitzer [32] discusses the utility of traces in relation to decisions about their analysis, we extend this concept here to trace collection, where investigators must similarly assess the potential value of a trace based on the information available during crime scene examination. When considering the potential implementation of rapid tests, their results may be used to inform these assessments of relevancy and utility at the crime scene. This could allow investigators to make more informed assessments of which traces are likely to add value to the investigation and are therefore relevant for collection, potentially resulting in more selective and targeted trace collection. As this may then lead to fewer traces being collected, it becomes even more important to understand how decisions are made at the crime scene.

### 1.3 Current study

Implementation of rapid testing into practice may prove beneficial as rapid test results can inform decision-making. However, the use of rapid tests is likely to interact with the cognitive processes involved in examining crime scenes. While guidelines and forensic science handbooks may help to standardize certain aspects of the crime scene examination, they provide little practical guidance for its cognitive dimensions. As a result, important decision-making processes may remain implicit, and rely on individual knowledge and experience that may vary greatly between investigators. This issue becomes particularly relevant when rapid tests are introduced, as they may allow for a stronger triage of traces at the crime scene. It is therefore essential to understand how crime scene investigators currently make decisions when examining crime scenes, as this may shape and help inform the future use of rapid testing, and how the availability and use of rapid tests may influence this. Accordingly, the current study aims to critically describe the literature on how crime scene investigators search for, interpret and collect traces; how they make decisions about subsequent trace analysis, and how the introduction of rapid testing may affect these different decision steps.

## 2 Methods

This literature review sought to investigate the cognitive processes underlying decision-making in crime scene investigations and to assess the potential impact of rapid testing on those processes. Given the broad nature of this topic, we used a scoping approach to the search for and selection of studies with broad search terms and inclusion criteria. However, decision-making processes at the crime scene have received limited attention in the literature. The review, therefore, focuses on studies addressing the search for, interpretation, collection, and analysis of traces, and the use and influence of rapid tests. For articles to be included, they had to (1) explicitly report how people in practice make or would make decisions regarding the approach of the crime scene examination (e.g., the search strategy), decisions regarding traces and their interpretation and (rapid) analysis, decisions regarding the overall interpretation of the crime scene; (2) report original empirical findings; (3) be written in English or Dutch; and (4) be accessible through open access or an institutional subscription. Both peer-reviewed or non-peer-reviewed articles were ultimately eligible for inclusion. Articles were excluded if they: (1) described purely conceptual or theoretical models of crime scene examination without presenting original empirical findings; (2) concerned processes occurring after completion of the crime scene examination that do not affect decisions made at the crime scene or the potential use of rapid tests; and (3) focused exclusively on technical aspects, such as preferences for a particular collection method, which fell outside the scope of this review. Trace analysis was retained within the scope, because the anticipated analysis of traces may influence decision-making at the crime scene (p. 21) [32], and because the decision to analyse a trace may reflect future decision-making regarding the use of rapid tests. Studies published up until the date of retrieval were eligible for inclusion. In addition, relevant studies published after this date were included if they came to the authors’ attention.

The identification of relevant literature consisted of several steps, starting with using keywords for decision-making^2^ and for crime scene examination^3^ to search the Web of Science Core Collection, Scopus, APA PsycINFO, International Bibliography of the Social Sciences, and Criminal Justice Abstracts (see Appendix A for the search queries and number of results). As this review focuses on the cognitive aspects of crime scene examination, databases were selected that include general, psychological and criminological literature and that were accessible through institutional subscriptions. The databases were searched on October 8, 2025, resulting in only English-language articles. In Scopus, English was applied as an explicit filter, because no Dutch-language results were identified, whereas several articles in other languages could be excluded. The Scopus search results were also limited to journal articles, as the search initially focused on identifying peer-reviewed articles reporting original results. These searches resulted in 911 records. As an initial screening indicated that relatively few records were relevant, the inclusion criteria were broadened to also include non-peer reviewed articles. Google Scholar was therefore searched on October 27, 2025, using the same keyword combinations as in the other database searches. A second Google Scholar search was conducted on October 31, 2025, using the term “mock crime scene” and combinations of keywords related to crime scene examination and trace interpretation.^4^ In addition, Scopus was searched again on August 25, 2026, with filters limiting the search results to conference papers, book chapters, and books. Ultimately, all search results from Scopus that may include original results were included. This led to a combined total of 1157 articles that were extracted, either through functionality provided by the database or through using reference manager Zotero (version 7.0.27), and combined into a single Excel file. To remove duplicates, all titles were converted to lowercase to harmonize formatting, and a new variable was created containing the DOI or, if no DOI was associated with the entry, the row number. Excel's functionality was then used to remove duplicates based on the lowercase titles and the new variable, resulting in 789 unique articles. These unique articles were manually screened based on their titles and abstracts, resulting in 79 potentially relevant articles.

The 79 articles that were not excluded during the first phase of screening were then read in depth. This resulted in the manual exclusion of 60 articles that either did not report empirical data on the decision steps examined in the review, were inaccessible, or did not report original results. Subsequently, forward and backward citation searches were performed on the remaining 19 articles to identify additional articles that had not been captured by the search queries. This process identified five additional articles and revealed that several included articles formed part of a doctoral thesis of which not all relevant chapters were published as independent articles. The doctoral thesis was therefore included in full. Furthermore, the authors became aware of three articles that had been published after the searches had been conducted. These articles were also included in this review as they met all inclusion criteria. Finally, Google's search engine was used on August 20, 2026, to identify additional (grey) literature, such as organisational reports.^5^ This resulted in the inclusion of three additional Dutch reports and one conference paper. See Appendix B for all 32 included articles and Fig. 1 for the inclusion flowchart.

**Fig. 1:**
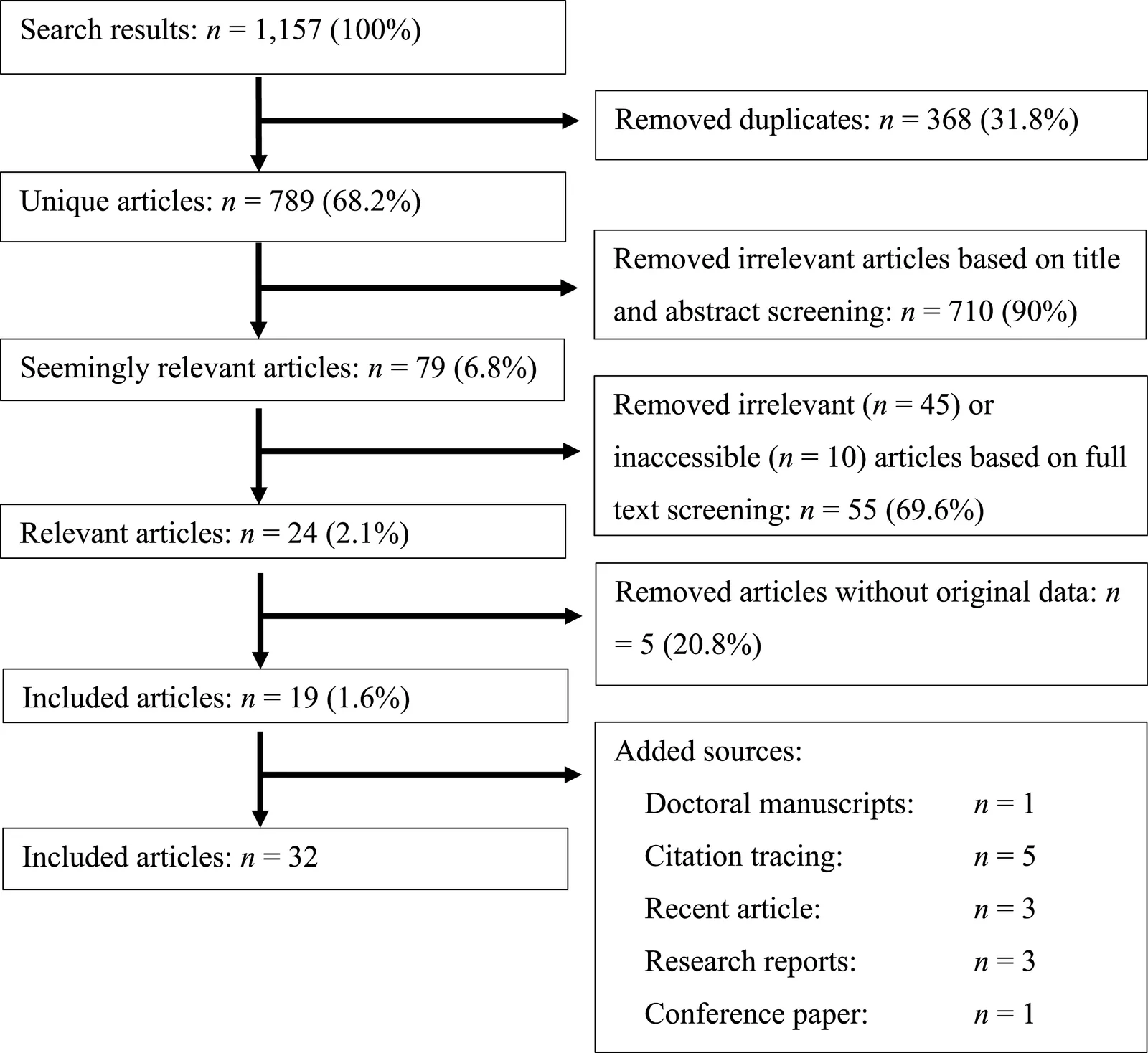
Flow chart of the results of the search results. *Note.* The percentages in the left column are relative to the total number of search results, while the percentages in the right column are relative to the number of search results before that step of exclusion.

## 3 Results

Recently, Divoy et al. [34] provided an overview of key decisions that comprise a forensic investigation, including decisions made at the crime scene. The current review builds on this overview by providing an in-depth examination of empirical findings about the cognitive aspects of decisions related to searching for, collecting, and analysing traces, as well as the practical influence of rapid tests on these decision-making processes. Given the limited insights offered by guidelines and handbooks, this review focuses on empirical studies, organised along the structure of the guidelines for crime scene examinations and the key decision steps identified by Bitzer [32]: starting information and first impressions, searching for and recognising traces, collecting traces, analysing traces, and the final interpretation of the crime scene, where each section will also discuss the potential impact from rapid testing. As research findings specifically on the influence of rapid testing are limited, we will use a narrative approach to synthesise the findings from current practices and findings specific to rapid test usage to hypothesise how rapid tests may influence decision-making at various stages.

### 3.1 Starting information and first impressions

The forensic investigation has already started before crime scene investigators arrive at the crime scene, as information is already being collected from the caller and uniformed police officers [35]. The information deemed relevant by the forensic team leader is then passed on to the crime scene investigators. Although this does not yet require a conscious decision on the part of the crime scene investigator, they do choose which information they actively seek out and which questions they ask colleagues already present at the crime scene. It does, however, seem unlikely that crime scene investigators can choose all information they receive, as the briefing from colleagues starts more or less immediately upon arrival and might be hard to interrupt [35]. Other investigators explicitly request more information, since they believe it can help them manage their expectations [36]. This can be problematic if investigators become overly focused on one explanation and disregard alternative explanations for the available information or their findings. This is particularly risky as forensic team leaders may fail to recognise how context information influences their decision-making [37], and may lack a thorough understanding of bias [35].

The influence of information received prior to the examination of the crime scene is demonstrated in an experiment where participants received either no information pertaining to the presumed cause of death or information indicating either a homicide or suicide before starting the examination of the crime scene [38]. Experienced crime scene investigators that received information indicating a suicide were subsequently more likely than expected to initially perceive the crime scene as that of a suicide. Conversely, crime scene investigators that received information indicating a murder were significantly less likely than expected to initially perceive the crime scene as that of a suicide [38]. Similarly, students in the murder condition wrote down murder significantly more often and suicide significantly less often than expected [39]. Information received before the examination may thus already influence expectations and, in turn, which traces are searched for and ultimately found. As the students had recently received training on bias but were nevertheless influenced by the prior information [39], it seems that preventing bias requires more than awareness alone. This is also supported by a more recent study, where warning students for overreliance on additional information did not prevent the provided information from influencing subsequent decision-making either [40].

#### 3.1.1 The influence of rapid testing on the starting information and first impressions

It could be hypothesised that whether rapid testing is likely to influence the available starting information and subsequent first impressions of crime scene investigators may depend on who will use the rapid tests. As discussed in 1.1, a rapid test indicating the possible presence of gunshot residue may be used to in- or exclude areas as part of the crime scene. Such a rapid test could potentially be used by uniformed police officers to determine whether a crime has likely been committed and if an examination is warranted. Although this potential shift in roles may depend on the accessibility of new techniques and may have far reaching practical implications for quality assurance and training, it seems plausible that rapid techniques may in the future be implemented in screening procedures for crime scene attendance.

### 3.2 Searching for and recognising traces

Due to the inherent uncertainty associated with crime scene examinations, crime scene investigators often do not know what to look for or which findings will ultimately prove relevant to establishing what has occurred. Guidance on the search for traces is also limited, as international guidelines and forensic science handbooks tend to focus primarily on systematic search strategies. However, because traces must first be recognised and interpreted as potentially relevant, and because not all traces are visible to the naked eye, systematic searching alone may be insufficient to find all traces. This was demonstrated in an eye-tracking study performed by Chang and Tsai [41], who found that the correct identification of key elements in a simulated crime scene depended on whether observers focused on key targets. Visible traces may thus be missed, and latent or otherwise invisible traces cannot be found using visual cues alone. Deciding where to search for traces, including visible, latent and other non-visible traces, is therefore crucial for finding traces and is essential for subsequent decision steps, since traces that are not found are lost to the investigation and cannot be used to inform further decision-making.

To find traces, crime scene investigators and students appear to reason about the modus operandi of the perpetrator [42]. This finding aligns with a narrative-based reasoning approach, where investigators form plausible scenarios about the activities that could have occurred and use these scenarios to guide their search for traces. Crime scene investigators have often been shown to use such reasoning [36], [42], [43], [44], [45], [46], much like forensic team leaders [37]. Narrative-based reasoning is, nevertheless, rarely mentioned in guidelines or forensic science handbooks. Although other scholars have suggested such an approach before (see for example [16], [31], [32] discussed in 1.2.1 and 1.2.3), little is known about how investigators construct these narratives in practice. Only Knes et al. [36] identified two strategies that crime scene investigators use to form scenarios. In the first, investigators use general observations to imagine different offender behaviours and hypothesise which activities might have occurred. In the second strategy, they form scenarios based on observed abnormalities that suggest potential offender actions.

Besides differences in personal preferences for the start of the examination [36], or time spent and paths followed [47], the narrative-based search for traces may further depend on the ability of the crime scene investigator to construct relevant stories. For instance, Watalingam et al. [48] conducted an eye-tracking study in which 37 crime scene investigators were asked to examine a mock crime scene. Of those investigators, fifteen fixated on a particular blood pattern caused by a gunshot, eleven commented on its significance, and only seven subsequently managed to use this information to find a bullet [48]. This illustrates that using trace information in the construction of stories requires reasoning about the possible causes of the trace and is not always self-evident. Although investigators with less experience displayed a similar pattern, more experienced crime scene investigators were on average more accurate in their reconstruction [48]. They also tend to devote a greater proportion of their time to important objects than trained novices or students [42], [48], which may be explained by experienced crime scene investigators having more detailed narratives about crimes and their resolution, allowing them to note more possible locations for traces. Baber [43], for example, compared experienced crime scene investigators with students in a small pilot study. He found that students focused only on areas they believed to be linked to stolen objects, whereas crime scene investigators also focused on the room's point of entry. Similarly, investigative psychologists were better at recognising behaviourally deviant cues than crime scene investigators [44], further illustrating how professional background and experience influence the recognition of traces.

Having more experience and relevant knowledge to drawn on may thus be beneficial if it contributes to the (correct) construction of the narrative of what happened [45], [48], [49]. Contextual information may similarly provide a framework for understanding the facts of a crime and support its reconstruction. Students that were asked to interpret a crime scene based on a photograph were, for example, supported by contextual information they received prior to viewing the photograph in focusing on the more factual elements of the crime [50]. However, expectations and contextual information that guide crime scene examinations can also negatively affect trace detection. For instance, Van den Eeden et al. [39] demonstrated that experienced crime scene investigators sometimes missed traces that students were able to find when examining a virtual mock crime scene. They hypothesised that this may have been due to the investigators’ stronger focus on objects with evidential value (i.e., expectations regarding which objects would be useful for the investigation, prosecution, and assessment of evidence), whereas students considered and examined a broader range of items.

Besides focusing on objects with evidential value, crime scene investigators may also be inclined to primarily focus on finding traces that can be used to identify suspects – traces with identification utility – rather than reconstructing what happened or testing hypotheses. An experiment conducted in the Netherlands, for example, suggests that Dutch crime scene investigators mainly searched for traces that were believed to be from the perpetrator and that only those traces were considered to be important [51]. This focus could, however, in part depend on the aim of the examination. Crime scene investigators may especially focus on finding traces that can be used to identify suspects when time pressure is high [47]. This may be the case for English crime scene investigators examining volume crimes as well [52], resulting in a context that is focused on efficiency and finding suspects [53]. Dutch crime scene investigators have, on the other hand, also been found to aim to reconstruct events while operating in a context of serious crime that is less focused on efficiency [53]. This aim may especially become prominent when a suspect has already been identified [47], or when crime scene investigators consider intelligence in their examination [54]. Although a focus on finding traces that can be used to identify suspects may thus be a deliberate decision to optimize efficiency, it poses the risk that other crime-related traces are overlooked, and that analysed traces and pursued leads are not actually related to the crime [51].

#### 3.2.1 The influence of rapid testing on the search for and recognition of traces

Although some rapid tests may theoretically be applied to entire crime scenes, it seems likely that most rapid tests will only be applied to specific traces or areas of interest. For example, a rapid technique that can illuminate lead particles may be applied to an area that is deemed relevant based on the constructed scenario to help crime scene investigators further determine where samples should be collected. When applied to specific objects, rapid tests may also support the assessment of the relevance of the object and help to recognise it as a trace. However, there is currently no empirical data on the influence that such applications may have on sampling behaviour. More generally, it could be hypothesised that rapid testing may influence the search for traces by shifting the focus of the crime scene examination. On the one hand, receiving an indication that a trace believed to originate from the perpetrator contains sufficient DNA for DNA profiling may shift the focus from identifying suspects to searching for traces that aid the reconstruction of the crime, as may happen in current practices when a suspect has been identified [47]. On the other hand, attention may shift to objects that could be analysed with the rapid test. The latter is supported by an experiment where crime scene investigators examined a mock crime scene in one of three conditions: having no additional tools; having access to a spectral camera that can be used to recognise and date bloodstains; or having access to a rapid identification test that can rapidly analyse and compare DNA-profiles and fingermarks to a database. Only crime scene investigators with access to the spectral camera seemed to attend more to objects with dark surfaces, although this did not lead to significant differences in the (crime-related) traces that were mentioned [51]. Further research is warranted to examine exactly how rapid testing could aid the construction of narratives to further guide the search for traces.

### 3.3 Collecting traces

After having recognised a trace as such, crime scene investigators need to decide whether the trace should be collected.^6^ It may seem reasonable to simply collect all traces, as instructed by the ENFSI [7] best practice manual, since uncollected traces will be lost. However, this may not always be feasible or useful. As an English crime scene investigator noted, collecting everything would cause you to grind to a halt, since everything would then have to be processed and recorded while 90% of it is unnecessary [52]. Moreover, an experiment in the Netherlands, where specific police regions could temporarily collect and analyse all potentially relevant DNA traces showed that the collection and analysis of additional traces did not lead to a significant increase in matches with the DNA-database [55]. Crime scene investigators must therefore, as described in 1.2.3, assess the expected utility and appropriate relevancy of the trace to determine whether the collection of an additional trace is warranted.

The number and type of collected traces appear to depend on several factors. First, features of the crime scene and offender behaviour influence the likelihood of trace transfer and persistence, influencing the likelihood of finding and collecting traces [56]. Second, available information and experience can influence trace collection. For instance, training in DNA and fingermark collection may lead to higher collection rates of DNA and fingermarks [57], while the availability of a report on behavioural cues can lead crime scene investigators to secure more deviant behavioural traces with high evidential value [44]. Similarly, experienced crime scene investigators collected far more DNA samples when examining a mock crime scene of a car than other actors from the criminal justice system [58]. Moreover, providing additional information about the success rates of sampling specific locations in the car for perpetrator DNA significantly increased the proportion of samples collected from locations with a high success rate, while decreasing the proportions collected from locations with moderate or low success rates. This information may, however, have diminished the influence of prior experience. The timing at which investigators received the information – before or during an initial round of trace collection – did not appear to affect this pattern [58]. Additional relevant information may thus help novices perform better, while potentially reducing the extent to which experienced crime scene investigators draw on their prior experience. Although finding more traces may not necessarily contribute to the assessment of expected utility and appropriate relevancy, additional information on success rates may be beneficial for this assessment.

Utility dimensions were, furthermore, considered when crime scene investigators decided on the collection of specific traces. Crime scene investigators from Sweden, Norway, and England, for instance, not only considered the interpretation of blood stain patterns but also the expected utility of traces for reconstruction purposes. In addition, they either attempted to collect as many different traces as possible or aimed to avoid mixed DNA-profiles [59]. The collection of a few high-quality DNA traces may then reduce the need to collect additional traces [36]. This aligns with the common focus on traces that can be used to identify suspects [47], [51], [52], [53], [54]. This focus might explain why investigators collected fewer traces when they interpreted the crime scene as that of a suicide, as compared to a homicide [39], although this finding was not replicated in a later study using different primes for homicide and suicide [59]. Finally, the number of traces collected seems to be unrelated to the time spent at a crime scene [47], but forensic investigators may collect more information when a relatively large amount of information is available, potentially to fill the last few remaining information gaps [37].

Crime scene investigators also appear to anticipate the later triage for trace analysis when deciding which traces to collect. When the number of traces that can be collected is limited, collection strategies may become more homogenous, with investigators prioritising traces that are more clearly related to the crime over more ambiguous traces [36]. This convergence in strategy under limited resources was further explored by Lidén and Almazrouei [59], who found that the number of traces that can be submitted for further analysis, as well as the availability of prior information, influenced which traces investigators collected. Limiting the resources seemed to decrease variation in collected traces between investigators who had received prior information indicating either a homicide or a suicide. In addition to limiting variation, resource constraints also seemed to lead to a different strategy for collecting traces compared to having unlimited resources. Instead of collecting fewer traces from the same locations, investigators chose to collect traces from different locations [59].

#### 3.3.1 The influence of rapid testing on the collection of traces

Although rapid tests can provide additional information about traces, potentially enabling crime scene investigators to better distinguish between relevant and irrelevant traces, access to rapid identification tests did not cause crime scene investigators to significantly differ in the number of collected traces when examining a mock crime scene [60], [61], although the control group without access to the rapid identification tests collected a specific blood trace more often (p. 93) [61]. This experiment did not, however, appear to assess whether traces that were initially collected would later appear irrelevant, and therefore not be taken to the police station, once rapid identification information became available. It is therefore important to explore whether and how rapid test results lead crime scene investigators to reconsider earlier collection decisions, including decisions about which collected traces should be transported for further analysis, and how the results can best be leveraged to optimize trace collection.

### 3.4 Analysing traces

Although triage for trace analysis may typically take place after the examination of the crime scene has concluded, it may nevertheless shed a light on the potential use of rapid testing as the same decision-making processes might apply. A principal consideration in the analysis of a trace is the available capacity for analysis, as this determines the need for selectiveness. Bitzer et al. [62], for instance, found that nearly all fingermarks and shoe marks could be analysed in-house for robbery cases in a region in Switzerland. Such traces were only excluded from analysis if they were of poor quality or redundant. They furthermore found that the results of prior analyses were also considered. If a suspect had already been identified, either through the forensic or tactical investigation, the analysis rate of biological traces dropped [62]. This suggests that crime scene investigators consider the utility of the results of trace analyses, which may explain why Swiss crime scene investigators were found to prioritise contact traces over “rich” biological traces. These crime scene investigators may assume that rich biological traces, such as blood, are more likely to originate from the victim and would therefore add less value to the investigation [62]. Prioritising contact traces instead may aid the reconstruction of the event, rather than just providing evidence against a suspect.

Triaging decisions are further informed by information, based on previous cases on which data is most promising in the context of a crime series or a typical phenomenon [54], or based on relevant scientific studies about success rates [58]. However, considerations of the perceived quality of the trace, personal experience and knowledge, and the nature of the trace appear to be more important in triage decisions [54]. A consideration of the perceived quality of a trace was also found by de Roo et al. [44], who found that some Dutch crime scene investigators base their selection of traces on the likelihood that the trace would be of high quality and suitable for comparative analysis. This also aligns with the common aim of identifying suspects [47], [51], [52], [53], [54]. However, after crime scene investigators were provided with additional behavioural information, they included significantly more deviant behavioural items and significantly less forensic items [44]. However, when relevant traces of good quality are unavailable, they also certainly turn to contact traces [62], for which it is difficult to predict the quality of a sample as contact traces are minimal traces that are typically not visible to the naked eye. Differences in findings might be explained by differences between regions and countries. Resnikoff et al. [54] studied six regions in Switzerland, of which only one was studied by Bitzer et al. [62], while de Roo et al. [44] studied Dutch crime scene investigators. Given the lack of guidelines or decision-support systems, it is not surprising that practices differ between countries and police regions.

#### 3.4.1 The use of rapid testing

The decision-making process surrounding the rapid analysis of a trace appears to at least partially overlap with current triage processes. As limited capacity for trace analyses necessitates triage, the availability of rapid techniques may expand capacity and, in turn, allow for less restrictive selection criteria during triage. Mapes (p. 97) [61], for instance, found that the availability of a rapid DNA-test led to more DNA-traces being analysed (including both rapid and laboratory testing) compared to the control group, even though there was no explicit limit set to the laboratory capacity. Similarly, the expected utility of traces also appears to influence the decision to use rapid tests. De Gruijter et al. [60], for example, found that a crime scene investigator did not submit a trace for a rapid identification procedure because the investigator believed to already have enough information. Like current triaging processes, crime scene investigators may also focus on identifying suspects as the rapid identification procedure in the examination of a mock crime scene was mainly used for traces that were believed to be related to the perpetrator [51], [61]. However, rapid identification tests can also be used to test hypotheses and exclude possible scenarios [63]. This may then increase the understanding of the crime scene and, as such, aid further triage. Furthermore, traces that were suitable for multiple analyses were often sent to a laboratory to maximize the information yield (p. 98) [61]. This also suggest that the crime scene investigators might not exclusively focus on identifying suspects but may instead aim to maximize trace utility. Regardless, it could be hypothesised that the focus may, like current triaging processes, shift away from identifying suspects when it is likely that a suspect has been or will be identified.

The quality of traces also appears to influence the decision to rapidly analyse a trace, much like current triage processes. Although success rates were mentioned less often than expected in the experiment conducted by Mapes (p. 98) [61], they were considered as a reason to analyse a trace when they were mentioned. Moreover, De Gruijter et al. [49] found that most Dutch crime scene investigators in their experiment selected traces with high success rates. Crime scene investigators may, however, not always be able to correctly estimate success rates. When asked to estimate the success rates of DNA-analysis on common objects, crime scene investigators from New York were found to have inaccurate beliefs, overestimating chances for objects with a low success rate [64]. Similar results were found among Dutch crime scene investigators (p. 100) [61]. In a subsequent vignette experiment among the crime scene investigators from New York, participants were asked to decide on the use of a rapid identification test either without information on success rates, with only information on success rates, or with both information on success rates and a decision support tool based on rational decision theory. Crime scene investigators only appeared to use the success rates when they were explicitly presented with them, and they only considered the serial nature of the case when they could use the decision support tool. Finally, no difference was found in the use of the rapid test between murder and burglary cases [64]. This may be explained by the experimental design where participants examining the burglary condition worked in the evidence collection team, mainly examining high-volume crimes (HVC), such as burglaries, whereas participants examining the homicide condition worked in the crime scene unit, which mainly examines cases involving a death [64]. A rapid identification device may, nevertheless, prove useful in both types of cases. For instance, a Dutch experiment in which investigators used a rapid DNA analysis system during criminal investigations showed that it was particularly valuable in HVC cases, as it resulted in the highest number of identifications in those cases [63]. Similarly, Mapes et al. [65] found that conventional DNA-analysis also led to more database matches being produced in HVC cases than in serious crime cases. This may be explained by the fact that perpetrators of high-volume crimes are more likely to have their DNA profiles stored in criminal DNA databases, thereby increasing the likelihood of obtaining a database match [63]. In contrast, a rapid identification device may for serious crimes be particularly useful for interpreting the crime scene, as it can help differentiate traces from different donors. This warrants further research into the considerations associated with the decision to use a rapid test and how the purpose of the rapid test may depend on the type of crime under investigation.

### 3.5 The interpretation of the crime scene at the conclusion of the examination

The final interpretation of the crime scene, after the search for and collection of traces has been completed, strongly depends on the earlier phases and decision steps, as they determine which information is available. This already starts before the crime scene is examined, as prior information may already influence expectations and the traces that are found. The experiment performed by Van den Eeden et al. [38], as discussed in 3.1, demonstrates that prior information pertaining to the cause of death can influence not only the first impression but also the most likely scenario after the crime scene has been processed. Although processing the crime scene led to more correct decisions, participants in the suicide condition were less likely than expected to correctly interpret the crime scene as a murder. This difference was, however, not significant [38]. A more recent study comparing English, Norwegian, and Swedish crime scene investigators with English laypersons similarly found that experienced crime scene investigators who received prior information indicating a homicide were more likely to judge the scene as a homicide than those that received prior information indicating a suicide, although the difference was again not statistically significant. The impressions and confidence judgements from English laypersons were, however, significantly influenced in line with the prior information [59]. The experiment conducted by De Roo et al. [44] where crime scene examiners were provided a behavioural report from investigative psychologists further suggests that bias may influence the final interpretation of a crime scene. Crime scene investigators who incorrectly interpreted the crime scene as that of a suicide were nevertheless very confident in their assessment and claimed not to need the additional information. As a result, none of those crime scene investigators changed their interpretation, even though the behavioural report could have led them to the correct decision as the investigative psychologists were significantly more likely to interpret the crime scene as that of a domestic homicide [44]. Regardless of bias, it appears that experts may be better at interpreting crime scenes than novices [48].

#### 3.5.1 The influence of rapid testing on the interpretation of the crime scene at the conclusion of the examination

Rapid testing may either directly influence the interpretation of the crime scene through providing additional information about traces or indirectly through the influence rapid testing may have on earlier decision-making and the resulting presence or absence of information. For instance, rapid identification results may help the reconstruction of more accurate scenarios as it can aid the interpretation of traces and lead to fewer other explanations [60]. However, correctly integrating the results into a final scenario may still prove challenging, resulting in only a small and non-significant difference between the number of correct elements in the final scenario after having examined a mock crime scene [60]. Similarly, the provision of rapid identification information indicating traces from at least two different donors did not lead English crime scene investigators to be more accurate in their assessment of the number of offenders [66]. The same experiment showed that the integration of rapid identification information may in part depend on the timing of receiving that information. Although crime scene investigators who received additional information about the donors of specific traces before they wrote down their initial scenario (the early group) were more often correct in their initial interpretation of the crime scene as a burglary leading to a homicide rather than a domestic homicide, the effect of the additional information was largest for the group that received the additional information after having written down an initial scenario (the late group) as nearly all investigators in the late group changed their scenario to a burglary. Investigators in the late group, therefore, more often arrived at the correct scenario. This may be explained by them being able to form their own interpretation and test this using the additional information, whereas the early group had a lot of information they struggled to integrate [66], [67].

Nevertheless, the fact that not all crime scene investigators changed their scenario in light of the new information, suggests that earlier beliefs may influence the interpretation of rapid test results. This may suggest that information is primarily integrated if the results align with the beliefs of the investigator [60]. Similarly, rapid identification results may result in crime scene investigators giving undue weight to traces, where traces that lead to an identification were considered to be more important than traces in a similar context that did not result in an identification [66]. This effect was only found among English and not Dutch crime scene investigators, which may be explained by a difference in the aim of the investigation. English crime scene investigators may be more focused on efficiently identifying suspects, while Dutch crime scene investigators may be more focused on reconstructing events [53]. It is therefore important to further study how rapid test results can best be integrated into scenario construction and how the influence of bias in this regard can be minimized.

## 4 Discussion

At the start of an investigation, many scenarios are possible and it is unclear which information or trace will prove important to the investigation. Crime scene investigators therefore examine crime scenes to identify traces that can help reconstruct events and potentially provide evidence for the prosecution of suspects. While guidelines and forensic science handbooks describe different phases of the examination and systematic search strategies, they offer little practical guidance on finding traces and decision-making. Similarly, empirical research into the cognitive aspects of crime scene examinations remains limited. Understanding how decisions are made at the crime scene is nevertheless vital for the potential implementation of rapid testing. The use of rapid tests depends on the traces that are found and collected, while rapid test results may also influence which traces are found and collected. Studies examining practical decision-making at crime scenes show that the search for traces is guided by narratives concerning the offender's potential activities. Potential traces are then collected and may be analysed if they appear to be of sufficient quality and sufficiently informative for the investigation. As such, the available information and personal experience play a major role in crime scene examination by informing search strategies and the assessments of the expected utility of traces. As a result, crime scene investigators may be biased by irrelevant or misleading information. The same risk applies to the use of rapid testing and may be even more pronounced when lines of inquiry, areas of investigation, or traces are excluded based on rapid test results. Preventing bias is therefore important to both current practices and the future use of rapid testing, as the crime scene examination is at the heart of the forensic investigation and its original state cannot be fully recreated at a later time.

### 4.1 Bias prevention

As prior information about a crime scene can already bias the subsequent investigation [35], it is noteworthy that there appear to be no current guidelines specifying which information should or should not be shared with investigators prior to their examination [35], [36]. Dutch forensic team leaders acknowledge the challenge of remaining open-minded once an incident has been labelled as a specific type of crime, but they still rely on the objectivity of investigators to mitigate the influence of bias [35]. Guidelines should therefore be implemented to ensure that information is communicated in a neutral way, and investigators should keep systematic records of the information they receive prior to and during their examination.

Dror and Kukucka [68], on the other hand, argue that crime scene investigators should not receive any contextual information before they have assessed the crime scene themselves. According to their ‘Linear Sequential Unmasking – Expanded’ (LSU-E) method, investigators should start with forming an impression solely based on their observations of the crime scene. Additional information may be introduced at a later stage, with the order in which it is provided determined by three criteria: its potential to cause bias, its objectivity, and its relevance. However, it remains unclear how this approach can be implemented in practice for examining crime scenes. Withholding potentially biasing information from crime scene investigators until they have documented their initial assessment might be impossible in practice, and it overlooks the potential benefits of having additional information for interpreting traces [50]. Furthermore, the LSU-E method does not provide guidance on how crime scene investigators should use additional information during their examination once it is made available [36], nor has the method been empirically tested among crime scene investigators.

Rather than initially withholding information from crime scene investigators, another approach emphasizes the consideration of alternative scenarios (see, for example, [16], [69], [70], [71], [72], [73]). This approach posits that findings should be evaluated by assessing how well they discriminate between plausible alternative scenarios. The NFI collaborates with law enforcement and academic partners to further develop this approach into practical steps that crime scene investigators can follow [74]. Their scenario-driven method for examining crime scenes aims not only to minimise the influence of cognitive biases but also to assist crime scene investigators in finding traces. While assessing the crime scene, investigators record their observations and use these observations to formulate scenarios about what might have happened. By identifying points of interests and key questions, crime scene investigators are guided in which topics warrant the development of scenes, or possible sequences of events. Crime scene investigators can then reason about the traces they would expect (not) to find if a given scene were accurate (see for a more detailed explanation [75]). For example, if investigators observe blood on a broken window, they might hypothesise that this was caused by the perpetrator being injured while breaking in. This could then prompt them to search for traces related to tending to that injury, such as in the bathroom. This is an iterative process, since any new traces found in subsequent searches can refine existing scenarios, lead to the creation of new scenes, and result in the detection of other traces. Considering alternative scenarios or scenes, while recording and explicating the observations and information on which these scenes are based, may make investigators realise that their beliefs about what happened could be mistaken and prompt them to search for traces that do not fit those beliefs. Additionally, this approach might not only combat the undue influence of bias, but also provide practical guidance to the use of rapid testing as the results could benefit the investigation if they can help discriminate between possible scenarios.

This scenario-based approach to examining crime scenes also appears to align well with current practices, since crime scene investigators already form stories about what happened. It is, however, critical that crime scene investigators also form alternative scenarios. Moreover, where the construction of scenarios currently seems to be based largely on experience and intuition, this approach structures scenario construction and aims to make explicit which information led investigators to consider specific scenarios. The efficacy of this approach, however, likely hinges on the investigators’ ability to construct actionable and relevant scenarios, a process about which little empirical evidence currently exists. Empirical research is therefore needed to investigate how scenarios, scenes, and search targets are constructed in practice, and to test whether this strategy effectively helps crime scene investigators identify more relevant traces. Similarly, it remains unclear whether considering alternative scenarios effectively reduces the influence of cognitive biases. Overall, findings appear to be mixed (see, for example, [76], [77], [78], [79]), and no empirical studies have yet examined these questions in the context of crime scene examinations or rapid testing at the crime scene.

### 4.2 The use of rapid testing

The introduction of rapid testing may substantially influence how crime scenes are examined. Information provided by rapid tests might lead to the inclusion or exclusion of specific areas of interest or traces, thereby affecting which traces are detected and which decisions investigators need to make. Rapid testing may therefore not only introduce an additional decision step, but also influence other decision-making processes throughout the examination. For example, the availability of a spectral camera may shift the search for traces towards surfaces for which the spectral camera is particularly useful [51]. Although such a shift in focus may facilitate the detection of certain traces, it may also result in other traces being overlooked. In addition, information provided by rapid tests may help crime scene investigators construct better informed scenarios [60], [66], [67], which may in turn lead to more effective search strategies. However, it is currently unclear under which conditions rapid testing benefits scenario construction and, consequently, the search for traces. Although rapid test results may also help to assess the relevancy and utility of traces and in turn benefit the triage for the collection of traces, further research is warranted to examine how rapid testing can optimize trace collection. Finally, the introduction of rapid testing moves the analysis of traces forward in the forensic investigation. As a result, rapid test results can be used for further decision-making, while also having the potential to rapidly guide the tactical investigation. Considerations for the rapid analysis of traces appear to largely overlap with considerations for current triage for trace analysis. Traces are mainly (rapidly) analysed when they are of sufficient quality and likely to be related to the perpetrator [44], [51], [54], [61]. Additional information, like forensic intelligence, information on success rates, or information that a suspect has already been identified, may further inform decisions about which traces are relevant for (rapid) analysis [49], [54], [58], [62], [64]. Future research should build on these findings by examining how test characteristics and case characteristics influence the use of rapid tests and the interpretation of their results. Such research could inform the development of a decision support system for rapid testing, potentially enhancing the transparency of decision-making [64].

### 4.3 Conclusion

Examining crime scenes contains a major cognitive component of interpreting traces, integrating information, and making decisions under uncertainty. These cognitive aspects are largely overlooked in the ISO-norms, ENFSI-guidelines, and forensic science handbooks. The practical guidance they offer remains limited to providing a sequence of activities and systematic search strategies. As a result, decision-making at the crime scene relies heavily on the available information, as well as the experience and knowledge of the individual crime scene investigator. Although personal experience may often benefit the investigation, reliance on such experience can make decision-making processes less transparent and less consistent. This becomes particularly relevant with the introduction of rapid testing. As rapid tests may give reason to include or exclude specific traces or areas of interest, they can directly influence which areas and traces are examined, and consequently which traces are found and missed. Similarly, discounting traces based on rapid test results may influence the interpretation of the crime scene and the corresponding search approach. This can, once again, influence which traces are found and missed. As empirical studies show that all these phases of a crime scene examination can individually be influenced, it can be expected that these influences permeate throughout the entire crime scene examination. The introduction of rapid testing therefore adds complexity to current conceptualizations of the crime scene examination, resulting in a less linear process and greater interdependence between the crime scene examination and trace analysis. The practical implementation of rapid testing may add yet another layer of complexity, as its use may be constrained by practical and organisational factors and may require a shift in responsibilities (see for example [63]), also resulting in the need for additional training and quality assurance protocols. It is therefore essential to further explore how rapid testing should be used to contribute effectively and appropriately to criminal investigations.

## CRediT authorship contribution statement

**Timon G. Udo:** Writing – original draft, Methodology, Investigation, Formal analysis, Data curation, Conceptualization. **Jasper J. van der Kemp:** Writing – review & editing, Supervision, Methodology, Funding acquisition, Conceptualization. **Christianne J. de Poot:** Writing – review & editing, Supervision, Methodology, Funding acquisition, Conceptualization.

## Declaration of competing interest

The authors declare that they have no known competing financial interests or personal relationships that could have appeared to influence the work reported in this paper.

## Figures and Tables

**Appendix A:** Search queries and results

Database	Query	Results
Web Of Science	(beslis* OR besluit* OR decision OR decisions OR decide OR "decision making" OR "decision-making" OR keuz* OR judg*) AND ("plaats delict onderzoek" OR "pd onderzoek" OR "PD onderzoek" OR "crime scene investigation" OR "crime scene examination" OR "crime scene analysis" OR "crime scene investigator" OR "crime scene examinator" OR "scene of crime officer" OR “crime scene”)	391
Scopus (Limited to articles)	TITLE-ABS-KEY ((beslis* OR besluit* OR decision OR decisions OR "decision making" OR "decision-making" OR decide OR keuz* OR judg*) AND ("plaats delict onderzoek" OR "pd onderzoek" OR "crime scene investigation" OR "crime scene examination" OR "crime scene analysis" OR "crime scene investigator" OR "crime scene examiner" OR "scene of crime officer" OR “crime scene”)) AND (LIMIT-TO (LANGUAGE, "English")) AND (LIMIT-TO (DOCTYPE, "ar"))	306
Scopus (Limited to conference paper, book chapter, and book)	TITLE-ABS-KEY ((beslis* OR besluit* OR decision OR decisions OR "decision making" OR "decision-making" OR decide OR keuz* OR judg*) AND ("plaats delict onderzoek" OR "pd onderzoek" OR "crime scene investigation" OR "crime scene examination" OR "crime scene analysis" OR "crime scene investigator" OR "crime scene examiner" OR "scene of crime officer" OR "crime scene")) AND (LIMIT-TO (LANGUAGE, "English")) AND (LIMIT-TO (DOCTYPE, "cp") OR LIMIT-TO (DOCTYPE, "ch") OR LIMIT-TO (DOCTYPE, "bk"))	154
APA Psychnet	(**Any Field**: beslis* *OR* **Any Field**: besluit* *OR* **Any Field**: decision *OR* **Any Field**: decisions *OR* **Any Field**: decide *OR* **Any Field**: "decision making" *OR* **Any Field**: "decision-making" *OR* **Any Field**: keuz* *OR* **Any Field**: judg*) *AND* (**Any Field**: "plaats delict onderzoek" *OR* **Any Field**: "pd onderzoek" *OR* **Any Field**: "PD onderzoek" *OR* **Any Field**: "crime scene investigation" *OR* **Any Field**: "crime scene examination" *OR* **Any Field**: "crime scene analysis" *OR* **Any Field**: "crime scene investigator" *OR* **Any Field**: "crime scene examinator" *OR* **Any Field**: "scene of crime officer" OR Any Field: “crime scene”)	0
IBSS	((TI,AB(beslis* OR besluit* OR decision OR decisions OR "decision making" OR decision-making OR decide OR choice OR choices OR keuz* OR judg*)) AND (TI,AB("plaats delict onderzoek" OR "pd onderzoek" OR "PD onderzoek" OR "crime scene investigation" OR "crime scene examination" OR "crime scene analysis" OR "crime scene investigators" OR "crime scene examiners" OR "scene of crime officer" OR "crime scene")))	23
Criminal Justice Abstracts	(beslis* OR besluit* OR decision OR decisions OR decide OR "decision making" OR "decision-making" OR keuz* OR judg*) AND ("plaats delict onderzoek" OR "pd onderzoek" OR "PD onderzoek" OR "crime scene investigation" OR "crime scene examination" OR "crime scene analysis" OR "crime scene investigator" OR "crime scene examinator" OR "scene of crime officer" OR “crime scene”)	191
Google Scholar 1	(beslis* OR besluit* OR decision OR decisions OR decide OR "decision making" OR "decision-making" OR keuz* OR judg*) AND ("plaats delict onderzoek" OR "pd onderzoek" OR "PD onderzoek" OR "crime scene investigation" OR "crime scene examination" OR "crime scene analysis" OR "crime scene investigator" OR "crime scene examinator" OR "scene of crime officer" OR “crime scene”)	92
Google Scholar 2	“mock crime scene” AND (“trace selection” OR “selecting traces” OR “trace interpretation” OR “interpreting traces” OR “trace prioriti?ation” OR “prioti?ing traces”)	0
**Total**		1.157

## Footnotes

1 A new standard for the recognition, recording, collecting, transport and storage of items is currently under development but has not yet been published [8].

2 beslis*, besluit*, decision, decisions, decide, "decision making", "decision-making", keuz*, judg*.

3 "plaats delict onderzoek", "pd onderzoek", "PD onderzoek", "crime scene investigation", "crime scene examination", "crime scene analysis", "crime scene investigator", "crime scene examinator", "scene of crime officer", "crime scene".

4 “trace selection”, “selecting traces”, “trace interpretation”, “interpreting traces”, “trace prioriti?ation”, “prioti?ing traces”.

5 The following search queries were used in this search: (1) “crime scene” AND “decision making”, and (2) “plaats delict” AND besluitvorming.

6 Crime scene investigators must also decide how traces should be collected technically, but this falls outside the scope of the current review.
